# Experiences of counselors participating in an innovative project to develop a training program for specialized foster parents of youth (13–18 years)

**DOI:** 10.3389/fpsyg.2023.1254700

**Published:** 2023-11-01

**Authors:** Pravin Israel, Jan F. Raae, Jone Ravndal Bjørnestad

**Affiliations:** ^1^Department of Social Studies, Faculty of Social Sciences, University of Stavanger, Stavanger, Norway; ^2^Faculty of Health Studies, VID Specialized University, Stavanger, Norway; ^3^The Norwegian Directorate for Children, Youth, and Family Affairs, Stavanger, Norway; ^4^TIPS–Centre for Clinical Research in Psychosis, Stavanger University Hospital, Stavanger, Norway; ^5^Department of Psychiatry, District General Hospital of Førde, Førde, Norway

**Keywords:** foster care, specialized foster care services, adolescent foster care services, training foster parents, child welfare services, bottom-up innovation

## Abstract

**Background:**

Children placed in foster care represent a vulnerable and distressed group that requires a high level of care. However, good training programs designed to address specific problems presented in specialized foster care are not easily accessible due to logistical, economic and structural barriers. The lack of easy access and a strong desire to provide high-quality services inspired counselors from a specialized foster care center on the frontline to initiate an innovative, developmentally relevant and locally grounded training program.

**Aims:**

This study focuses on counselors’ experiences with the development of the training program and its impact on their work life.

**Method:**

A qualitative research design within a participatory approach framework was used to understand the experiences of the counselors. All the counselors employed in the department and the leaders (*n* = 14) participated in the study. Data were gathered from participants, including the lead and second authors, using a semi-structured interview, and analyzed using thematic analysis.

**Results:**

The analyses yielded three main categories: (i) Psychological Factors, (ii) Social Dynamics, and (iii) Leadership Style and Support. Each of the first two categories consisted of three subcategories. At the psychological level, the employees’ experiences reflected the psychological states and traits required to fuel the tasks required by the project. The social dynamics of working in a team influenced the work process and, in turn, were impacted by it. Lastly, leadership style and support provided the foundation for innovation to germinate and grow.

**Conclusion:**

Engaging in a locally created training program was associated with a strong sense of collaboration and team spirit. Counselors reported high intrinsic motivation and a strong sense of personal pride and drive for their jobs. They were proactive in seeking colleagues with particular expertise and collaborated on project tasks despite differences. The leadership style reflected the presence of transformational leadership behaviors, signaling an organizational culture conducive to innovation. The study provides an example of how aligning employees’ personal aspirations with workplace goals and professional development can create a workplace in which employees feel it is enjoyable to go to work.

## 1. Introduction

Children placed in foster care are a vulnerable and distressed group (Bruskas, 2008; Engler et al., 2022; Straatmann et al., 2022; O’Hare et al., 2023). In response to their needs, private and public agencies provide foster home services where foster parents are recruited and trained to provide a home environment to mitigate the ill effects of the children’s negative experiences and promote healthy development. However, some children who are very distressed and traumatized warrant protracted care provided by adults with a high-level of knowledge and skill. Residential institutions employ experienced professionals to provide a safe environment. Specialized foster care (SFC), which provides care in the context of a family, especially older children and adolescents, has proven to be a viable alternative to institutional care (Webb, 2010; Åkerman et al., 2023; Chartier and Blavier, 2023).

Training programs for foster parents span from mandatory pre-service programs, which have recruitment as the primary agenda, to in-service programs that target providing knowledge and skills relevant to the foster parent’s particular situation and need. For instance, the Norwegian Directorate for Children, Youth and Family Affairs (Bufetat) provides pre-service training (Spesialisert fosterhjem, Bufdir, 2023). Advanced in-service training programs targeting specific knowledge and skills required for a particular behavioral or emotional problem have been developed, tested, and documented favorable outcomes (Solomon et al., 2017; Cooley et al., 2019; Åström et al., 2020; Cooley and Krysik, 2022; Lassmann, 2023). The specialized programs are embedded in the larger context of the national public welfare service (Ogden et al., 2008; Spesialisert fosterhjem, Bufdir, 2023; Treatment Foster Care Oregon (TFCO) – NUBU, n.d.). Nonetheless, the programs are not widely disseminated, and access to frontline services is limited due to structural restrictions and the costs associated with training and certification.

In recent years, trauma-informed care (TBO) has been available to foster parents and counselors in Norwegian public welfare services (Steinkopf et al., 2020). Although the principles of TBO can inform and provide some guidance, it lacks the targeted focus on knowledge and skills to address and resolve the problems of foster parents and children in SFC (Spielfogel et al., 2011). There is also a general lament that high-quality evaluation of foster parents’ training programs needs to be improved (Festinger and Baker, 2013). Solomon et al. (2017) conducted a meta analytic study assessing two effect levels. The first level assessed the direct effect of the training program on the level of knowledge and skills of the foster parent. The second level examined the effect of the training program on child outcomes. The results were promising for both outcomes. However, the authors noted significant methodological weaknesses, such as the lack of a control group, thereby endorsing Festinger and Baker’s (2013) conclusion that high-quality evaluation of foster parent training programs is lacking. Still, training programs like MAPP (Cooley and Petren, 2011) and PRIDE (Nash and Flynn, 2016; PRIDE Model of practice overview – CWLA, n.d.) that lack a theoretical foundation and high-quality outcome studies are widely disseminated and used in foster care services.

Counselors working with specialized foster parents have a close and complex working relationship, often balancing the demanding realities of therapeutic engagement with foster parents and children and system navigation (Fulcher and McGladdery, 2011; Brown et al., 2015; Frieiro Padín et al., 2021). For example, counselors are commissioned to provide a variety of services to foster parents. They provide personal support to foster parents. For instance, foster parents may themselves have adverse childhood experiences and traumas that are exacerbated after taking in a foster child (Adkins et al., 2020; Mancinelli et al., 2021; Reisz et al., 2023). Foster parents report that managing their children’s behavioral and emotional problems and dealing with biological parents are among the most demanding tasks, but they lack access to specialized training programs. Counselors also help foster parents manage their biological families. The biological children of foster parents report experiences of lack of involvement in the decision to become a foster home and feel marginalized and invisible (Possick et al., 2022). Lack of counselor support can interrupt smooth functioning and result in placement disruption (Vanderfaeillie et al., 2018).

Additionally, counselors must deal with system-level hurdles such as translating policies into actionable tasks and implementing them into practice. The process is complex, and counselors often face dilemmas that challenge their ethical code, feasibility, and pragmatic implications (Tummers et al., 2015; Trappenburg et al., 2020). Although counselors play a crucial role in the success and quality of foster care services, they are exposed to demanding work conditions (Smith, 2005; Li et al., 2020). Studies show that lack of engagement, emotional exhaustion, cynicism, professional efficacy, and leadership satisfaction predict the intention to quit. Conversely, low conflicting demands, high level of openness, high degree of perceived service quality, and continued experience of professional development predict intention to stay (Clark et al., 2013; Astvik et al., 2020; Nilsen et al., 2023).

Faced with the challenges of accessing relevant technologies and yet inspired by a desire to provide high-quality services and pursue meaningful professional development, counselors in a specialized foster care department in Norway took on an innovative project. The primary objective of the present study is to evaluate the counselors’ experiences of participating in this project and to understand its impact on their professional lives.

## 2. Methods

The current study used a participatory approach to actively involve all participants in the development of an innovative training program and evaluate their experiences. We initially considered using Interpretive Phenomenological Analysis (IPA) as a research design, but ultimately chose Thematic Analysis (TA) proposed by Braun and Clarke (2021) for several reasons. First, our main objective was to identify patterns and themes among all participants rather than to gain a deep understanding of only a few. Second, the project was designed with a collaborative framework for participants to have a high level of involvement. Lastly, we wanted a flexible methodology and TA provided the most comprehensive understanding of counselor experiences and attitudes.

### 2.1. The study context

The Norwegian Child Welfare Services are organized at the municipal level and state level. Specialized foster care (SFC) is responsible for adolescents (13–18 years) and is organized at the state level. SFC services in Stavanger, Norway, consist of two departments under two organizational streams. One department (Family Homes, Stavanger-FHS) is organized with two other departments in southwestern Norway, consisting of 7 counselors and a department head. Another department (Stavanger Youth Center-SUS) is organized under the residential youth services in Stavanger and consists of 4 counselors and a department head. The department heads had managerial responsibilities for all the employees involved in the project and did not participate in the development of the content for the project. A clinical psychologist (the first author) ensured the overall quality and professional competence of the services.

Henceforth in the article, we shall refer to both departments as the Stavanger Department. The Stavanger Department has about 28 specialized foster parents (henceforth known as foster parents) in the region.

### 2.2. The innovative project

The first author joined the Stavanger Department as a clinical psychologist in the fall of 2021, taking on various responsibilities including the professional development of counselors and quality assurance. Shortly thereafter, the counselors expressed a need for a relevant and accessible training program for the foster parents they supervise. Leveraging the first author’s expertise in developing and implementing clinical interventions, the department initiated a project to create a locally-anchored training program for foster parents. The first author provided leadership to the project.

The overarching goal of the project was to develop a training program that was both developmentally relevant and rooted in the local context of Stavanger, Norway. The program’s main innovation lay in its collaborative development process, which actively engaged and incorporated: (i) the practice-based knowledge of counselors and leaders, (ii) the user experiences of foster parents, and (iii) the research-based insights provided by the first author (also see O’Cathain et al., 2019). This development was integrated into the regular workflow, using the time left for professional development.

All participants, hereafter referred to as the project group, participated in regular group discussions about, among others, their experiences in supporting and supervising foster parents. A recurring theme was the need to educate foster parents to better understand adolescents (ages 12–18) in foster care. Consequently, the project group agreed to develop a training program consisting of three modules that addressed the foster parents’ needs for a systematic understanding of (i) normal developmental changes in adolescence, (ii) disruptions in normal development and its consequences, and (iii) self-care for foster parents and their families.

Each module was structured to include four subtopics, each lasting one hour, allowing for the entire module to be completed in a single day. For example, the objective of Module 1 was to provide a foundation for understanding developmental milestones in adolescence. Its four subtopics were: (i) Understanding Adolescence as a Developmental Phase, (ii) Biological Changes in Adolescence, (iii) Psychological Changes in Adolescence, and (iv) Social Changes in Adolescence. Modules 2 and 3 had similar structures, each with their own specific goals and subtopics.

The project group was divided into four teams and given the responsibility of developing a given subtopic. The first author provided ongoing support and supervision to the teams during this development phase. Upon completing the subtopic, and consequently the module, the project group conducted a preliminary workshop and presented their subtopics to the foster parents. They requested feedback on content, presentation style, and potential improvements. Two modules were completed in a similar fashion, and Module 3 is still under development. The current study was conducted after Modules 1 and 2 had been presented to the foster parents.

### 2.3. Participants

The 14 participants (10 female) in this study represent all the employees in the Stavanger Department. Participants had an average of 23 years (range 7–35 years) work experience with children and adolescents and an average of 5 years (range 0–23 years) work experience in the Stavanger Department. The professional background of the employees was as follows: 7 social workers, 4 child welfare workers, 2 with diverse social backgrounds, and one clinical psychologist. Most counselors had advanced training in family therapy (*n* = 4), milieu therapy (*n* = 2), social competence (*n* = 2), public administration (*n* = 1), and clinical child psychology (*n* = 1).

### 2.4. Recruitment and data collection

The project started in Fall 2021 with only Family Home, Stavanger (FHS) as the participating department. The second department, Stavanger Youth Center (SUS), joined in the Spring of 2021. The evaluation by the counselors was discussed at the inception of the program as part of the internal quality assurance work. Furthermore, although it was not required formally, all participants in this study gave their informed consent. All counselors were directly involved in the program’s development; therefore, participants in this study represent the complete sample. The first author interviewed the counselors and the two department heads in Spring 2022 (average 37.08 min; range 23.24–45.09 min). Thereafter, one of the counselors (the second author) interviewed the first author (37.19 min). All interviews were recorded and transcribed verbatim for further analysis.

### 2.5. The semi-structured interview

A semi-structured interview was fashioned to elicit tacit knowledge gained from the practice experience (Welsh and Lyons, 2001) and generate rich data suitable for thematic analysis. The questions that guided the interview were designed to extract themes across the entire sample group rather than in-depth themes with only a few participants. This strategy allowed for a broader understanding of attitudes, experiences, and impacts related to the project, and ultimately on the counselors’ professional roles. The interview questions were as follows: (i) Until recently, how have you worked with the foster parents to keep them abreast with the relevant professional area? (ii) Can you talk about your attitudes and experiences regarding participation in the project in the department? (iii) What has the work with the project done to you? and (iv) Do you have any other comments and/or suggestions regarding the professional development project at the department?

### 2.6. Researcher roles and biases

The first and second authors worked for the Stavanger department, which presented challenges in maintaining confidentiality and avoiding biases such as social desirability bias, confirmation bias, and observer bias. To address these issues, the authors took several measures to encourage honest and open responses from the interviewees. The interviewees were instructed to focus on the process and impact of their involvement in the project and to be mindful of their responses. However, there are inherent limitations when interviewing colleagues, as respondents may censor or moderate their responses, leading to incomplete or inaccurate data. A mitigating aspect, as the department psychologist conducting the interviews, the interviewer was bound by professional guidelines for confidentiality, which helped establish a trustworthy environment. Additionally, the second author interviewed the first author to introduce objectivity and reduce power dynamics. To further mitigate observer and confirmation bias, an external scholar (third author) was incorporated into the coding and thematic development process to serve as an external validator during the data analysis phase.

### 2.7. Data analyses

After completing the interview, all participants were invited to transcribe the interview themselves and provide *post hoc* additions if necessary. None of the participants used this option. With the exception of an interview, the data transcription was performed by a professional company. The second author interviewed the first author and transcribed the interview verbatim. All other interviews were verbatim transcribed by a professional company and imported into the Nvivo 11 software package for further analysis. Data were analyzed using the thematic analysis procedures recommended by Braun and Clarke (2021). The three authors were involved in the coding and thematic analysis. First, they individually coded the categories based on the concrete steps presented in Table 1. Thereafter, they interacted with each other to arrive at the final coding, guided by the following principles: (i) parsimoniousness (i.e.*, when two explanations seem right, the more straightforward explanation is preferred*) (ii) minimal deviation from the actual descriptions in the raw data, and (iii) choosing the best argument.

**Table 1:** Steps of the thematic analytic strategy.

Step	Title	Description
1	Familiarization with the data	Immersion in the data by reading and re-reading the entire dataset to gain a holistic understanding. Take initial notes and identify patterns or interesting points.
2	Generating initial codes	Identify meaningful units of information in the dataset and assign descriptive codes to them. These codes should capture the essence of the data.
3	Searching for themes	Examine the coded data for potential themes. Look for patterns, connections, and relationships between the codes. Themes are recurring patterns of meaning within the dataset.
4	Reviewing themes	Review and refine the initial themes by comparing them with the dataset. Ensure that the themes accurately represent the data and have internal coherence. Adjust and modify themes as needed.
5	Defining and naming themes	Clearly define and describe each theme concisely. Develop informative and representative names for each theme that capture their essence.
6	Creating an analytical narrative	Craft an analytical narrative that weaves the themes together, illustrating the main findings of the study. Use supporting quotes and examples from the dataset to provide evidence for each theme.
7	Ensuring rigor	Engage in critical reflection and check the analysis for its trustworthiness. Seek feedback from colleagues or peers to ensure the validity and reliability of the findings. Document the decision-making process.

### 2.8. Ethical issues

The project was classified as a quality assurance initiative and did not require formally consent. However, ethical best practices were followed. No identifiable and personal data (as defined by the Regional Ethics Committee) were collected. Additionally, to guide participants through the research procedures, written information was provided on the project, the voluntary nature of the interview, confidentiality, and anonymity in presenting the results. Consequently, in response to these procedures, all participants gave their consent. The quotations in the article are presented anonymously.

## 3. Results

Analyses yielded three main categories and a total of six subcategories. These categories and subcategories are presented in Table 2.

**Table 2:** Categories and subcategories.

Main category	Sub-categories
1. Initiating and fueling the innovation	i. Unfamiliar but Exciting ii. Serendipitous Alignment iii. Intrinsic Motivation
2. Interpersonal dynamics	i. Fostering collaborative support ii. Managing interpersonal conflict iii. Team dynamics
3. Leadership style and support	

### 3.1. Initiating and fueling the innovation

The main category focuses on the conditions necessary to initiate and sustain innovation in the public sector. Three subcategories emerged from the data (i) Exciting and stimulating, (ii) Serendipitous Alignment, and (iii) Intrinsic motivation.

#### 3.1.1. Unfamiliar but exciting

All the counselors in the project are involved with various tasks related to supervising and supporting the foster parents. However, the project presented counselors with a new task: designing, forming, and teaching foster parents concrete topics related to the target group, that is, adolescents in foster care. On the one hand, the counselors were optimistic and excited, but some were also cautious. One counselor reported that *“we got to focus on something that we did not do earlier, namely, to produce professional content that was concrete, useful, visible, and something we could use…this produced positive ripples individually and collectively, I think* (C_09). Another recollected that “*the project was exciting, and the employees had the knowledge and experience but lacked theory…and some did not stand in front of people, so that brought forth many feelings* (C_12)*.”* In terms of the challenges the counselors experienced, they did not seem deterred but saw it as an opportunity to learn new skills and grow. Counselor (C_01) reported that “*there were many details regarding how to use my voice and formulations which I got to practice. And I was not so afraid, really, to stand in front…but I really wanted to present something that is good, and this is something all the counselors can identify with…so an unfamiliar role, but alright”.* Another learning-related skill that the counselors reported was “reflexivity.” The ability to reflect on one’s performance and learn from it is an important factor in learning. One of the counselors (C_02) discussed the professional role and responsibility in developing the course by emphasizing that it had to be “*safe and yet understandable. Further, there should not be any misunderstanding.”* Rewards directly enhance learning, and a counselor said that “*the bosses actually got to see that we have a good deal of knowledge and can accomplish… be seen* (C_03)*.”* One counselor summarized the project by saying, “But *all in all, it has been demanding and rewarding for the department* (C_12).”

#### 3.1.2. Serendipitous alignment

Since the counselors have been working in the field for many years, their work experience primed various targets for development. The innovation allowed them to recall their personal aspirations and draw upon their strengths. One counselor felt thrown into the deep end of the ocean, but was not shaken because she was confident that she could do the job, demonstrating openness and adaptability. There were also other reports of serendipitous alignment of their long-term personal goals with the project’s goals: “*I was in a way searching to find the norms and knowledge and thankful for all the knowledge…for those who have worked for a long time and found their methods, I think it may not necessarily be that the safest route to the target is the best one.* (C_08)*.”* Furthermore, *“the theme is something I have been burning for and have been interested in for many, many years. So, I think it was very exciting to go deeper into that…*(C_03).” These quotes from counselors show that through their work experience, they developed deep and personal aspirations for work over time. The innovation opened these fundamental long-term personal aspirations to surface and fuel the work.

#### 3.1.3. Intrinsic motivation

The innovative project work came on top of the routine duties of the counselors. Although such tasks over time can lead to detrimental outcomes for employees, the counselors reported an intrinsic motivation to focus on the project’s tasks. One counselor recalled*, “It was busy in my usual job, such that it became demanding. However, one performs better when pushed, and when one pushes themselves…it was exciting academically and professionally, and everyone stretched themselves a little…in that way, it has been an activation of academic and professional resources in each of us* (C_01). The intrinsic motivation to initiate and continue the work was also seen as a challenge to “*show him (the project leader) what they could read and produce…I have seen the workplace operations and academic focus in a way that I have not seen before I think* (C_04).” However, it took time for the counselors to start experiencing the motivation to kick in. When it did, the project positively impacted the counselors: “*True, well, when it had started to sink in, and we were in motion, everyone was in motion, and we could start breathing again and maybe relax our shoulders a bit. Moreover, it* var*ies from person to person, so I think that this gave energy, and it gave a sense of accomplishment, it gave a feeling of having something* (C_09).” Finally, a counselor’s testimony to the inspiration the project provided was as follows: *“I have been looking forward to going to work, I have found it enjoyable… So, for me, it has been extremely positive, it has made this year, in a way or half… Three-quarters of the year, it has been enjoyable to go to work* (C_03)*”*.

### 3.2. Interpersonal dynamics

Addressing how the team works and its interpersonal dynamics was unavoidable in assessing workplace innovation and something the counselors discussed. There were three subcategories of (i) Fostering Collaborative Support, (ii) Managing interpersonal conflict, and (iii) team dynamics.

#### 3.2.1. Fostering collaborative support

A salient point that illustrates collaboration was that counselors sought out colleagues with a special interest and competence in a particular area and enlisted their help or used them as sparring partners. There was recognition that such a strategy would secure a better basis for deciding on the design and content of the themes in the project. Additionally, counselors felt that this collaboration inspired them to work more and find effective ways to present the topics to foster parents. One counselor illustrated active collaboration as inspiring and said, “*Soon (after talking to the colleague) you want to find out more and you catch yourself returning to your workstation and starting the process of retrieving more information* (C_08).*”* However, only some of the counselors felt that there was a culture of active collaboration during the project period. Sometimes, they felt alone and could not collaborate with a colleague with whom they were supposed to partner on a particular task. However, there was a sense of understanding and accommodation of the shortcoming that they experienced. There was also a clear understanding that feeling seen, heard, and accepted by others in the team was necessary, as demonstrated by a counselor who said, “*We did not have opposing opinions, but we approached the topic from very different starting points, which was important. I have learned a lot from my collaboration with (a particular colleague) …*(C_02).” Diverse ways of approaching a said goal and working through it despite very different approaches were complemented by the distribution of the various tasks within the team: “*I think perhaps that some people in this group could have been good at doing the research and finding the material and such, then there could have been others who received the material, familiarized themselves with it, and were good at lecturing it* (C_04).”

#### 3.2.2. Managing interpersonal conflict

There were pre-existing conflicts that counselors discussed in the context of how they experienced the workplace before the project’s onset. A counselor recalled that the work environment was often difficult and that much energy was invested talking to each other, seeking support, and alleviating frustrations: *“The work environment has been poor. And I’ve had a very okay time with some people, but overall it has affected… Much time has been spent sitting with each other, seeking alliances, sort of like investing a lot of energy into going to an office and expressing your frustration both in one direction and the other” (C_03)*.

Another counselor reported that she dealt with the situation by being polite and courteous, and focused on the tasks at hand without more involvement with other colleagues. One counselor reported that the conflict had stabilized. There was a conspicuous lack of conflict or disagreement related to the project, and all participants worked to accomplish the project’s goals. The project appeared to have given a common purpose, and people worked on their specific tasks and accommodated the others in the team when active collaboration was less than ideal, and one could not contribute as much to specific areas of the work: “*Due to illness at home, I worked independently a lot, missing some collaborative meetings. My colleague had reduced hours… despite our different perspectives, collaborating with her has been a valuable and positive learning experience for me”* (C_02).

#### 3.2.3. Team dynamics

One of the benefits of the project was that the counselors got to know each other better. They described a positive work environment that accentuated their sense of group cohesion: “*While we had this project, I got to know many other people, so it’s more enjoyable to walk around the hallway here afterwards. Because I know everyone’s names and I know a bit about what they are interested in and what strengths they have. And then it’s also easier to sort of cheer them up and support them a little and…*C_08).” Furthermore, since the project involved two departments, the project provided them with the opportunity to get to know each other and foster cross-departmental relationships. There was a feeling of comradery that developed as a result of working with colleagues who were new employees and colleagues from a different department: “*In the group, I have been working with someone who is newly hired and someone from the other department… it has been very interesting to get to know them and see what they stand for and how they work and… Yeah, involve them. So, I think it was good*. (C_03).” Furthermore, another observed that “*the counselors were engaged in the tasks, applied themselves as a group, and challenged each other to go beyond their comfort zone. There was a sense of security in the team and they felt cared for* (C_12).” Counselors acknowledged that the project also had a positive ripple effect upward in the organization and were happy on behalf of the leadership. They noticed that the department leader was proud and happy and could boast of the project to her colleagues and other leaders in the organization. The counselors acknowledged and supported the efforts of the leaders and their colleagues. Team dynamics was affected by the positive work environment and, in turn, was impacted by it.

### 3.3. Leadership style and support

Leadership style and support were crucial in initiating, implementing, and sustaining the innovative project. The leadership at the department and even higher levels were proactive, supportive, and encouraged the development and implementation of the innovation: *“and I think the leaders were very excited about the project. There was unconditional acceptance… and even the top-most leader gave her positive support* (C_12).” There was also the notion among the leaders that since the team had not been involved in systematic professional development, this project presented a unique opportunity, and they were therefore on board. The message that “I am here to help and support” came from several levels of leadership and facilitated buy-in and onboarding. However, there was also the sentiment that leadership support was contingent on one’s role in the system; “*I think first and foremost about participation, and then I think that the psychologist has a little more clout in both departments to say that we should do this, and we should set aside time for this* (C_08).” Large public organizations are known for their hierarchical structures. However, leaders who actively participate and allocate resources to innovation efforts can significantly impact and lead to successful innovation. Reflecting on the process, the project leader felt that she gained the trust and acceptance of the team, which made it possible to develop and implement the project. In addition, counselors appreciated the proactive and supportive role of leadership and highlighted their importance in creating an environment conducive to innovation.: “*I think it is great. I think it is very. Very good. And I think it has been good for the group regardless of the degree they have worked with it*” (C_11).

## 4. Discussion

The aim was to examine the experiences of the employees in a specialized foster care service center with an innovative project developed and conducted by the employees. The study highlighted how psychological and social factors and the structural dynamics of a workplace located in a public welfare sector impacted employees and the workplace. The analyses yielded three main categories and six subcategories. At an individual level, the employees’ experiences reflected the psychological states and traits required to fuel the tasks required by the project. The social dynamics of working in a team influenced the work process and, in return, was impacted by it. Lastly, leadership style and support provided the foundation for the innovation to germinate and grow.

### 4.1. The psychological factors

The experiences of the counselors within the project resonate with the established literature on the conditions necessary to successfully implement workplace innovation. The principles of learning and reflexivity, coupled with extrinsic rewards such as visibility and recognition, were critical to the project’s success. Reflexivity, a key component of professional development, was particularly significant, as it allowed counselors to engage in introspection and self-evaluation, despite the demanding nature of their roles (Illiuschenko et al., 2021). The project’s ability to challenge counselors beyond their comfort zones, a known factor that improves effective learning (Dornan et al., 2019), further facilitated this process. This aligns with the literature on coping (Lazarus, 1993; Piao and Managi, 2022), suggesting that feelings of accomplishment and self-evaluation often arise from overcoming challenges beyond one’s perceived capabilities.

The counselors found a welcome match between the tasks of innovation and their personal long-term aspirations for the job. This finding is significant for two reasons. First, when there is a strong alignment between personal aspirations and work-related tasks, they will likely be more engaged and motivated (Locke and Latham, 2004). Sometimes, the inspiration and desires of counselors, over time, become buried under uninspiring routines and tasks. Innovations that allow them to expand their knowledge and skills, which were previously motivating, will likely produce renewed excitement for the job and increase the chances of success. Secondly, the ability of employees to maintain knowledge and skills over many years and reapply them is associated with psychological traits such as openness to experience, adaptability, and conscientiousness associated with long-term goal setting (Barrick et al., 2013). Holding on previous knowledge and skills that are not readily visible due to a lack of opportunities in the workplace is associated with a loss of motivation and a sense of being redundant. This observation brings to sharp relief the need to create opportunities and engineer situations for counselors’ personal aspirations to align with the innovation, in other words, a leadership challenge.

Our counselors reported a high intrinsic motivation to engage with the innovation despite their regular workload and routine duties. The reason for enhanced motivation was likely because of meaningful goals and tasks that required them to stretch themselves academically and professionally. However, finding meaning in goals and tasks can take time, as an informant suggested, and requires forethought as to how motivation can be lifted in the early stages of the project. This finding aligns with the literature suggesting that intrinsic motivation, as a psychological state, is a vital driver of workplace innovation (Ryan and Deci, 2000; Bawuro et al., 2019; Itri et al., 2019). Since psychological states are time- and context-bound and presenting challenging work goals and tasks keeps up the flow of motivation, herein is a condition that is necessary to keep the wheels of innovation moving.

### 4.2. The social dynamics

The counselors’ experiences in the innovative project underscore the intricate interplay of fostering collaborative support, managing interpersonal conflicts, and nurturing positive team dynamics in driving workplace innovation. For example, counselors voluntarily sought out colleagues with specific interests and expertise and used them as sparring partners. Such an initiative embodies the team spirit and contributes to the success of the project’s outcomes. Additionally, this finding aligns with research suggesting that collaboration and knowledge sharing are key drivers of innovation (Hülsheger et al., 2009; Alexander and Childe, 2013). However, the results also showed that collaboration could be hindered by factors such as lack of time, conflicting priorities, and differences in work styles.

Another essential feature that our study confirmed was the importance of being seen, heard, and accepted by other members of the team. Psychological safety is crucial in innovative project work, as understood by the shared belief that the team is a safe environment for adventurous people to take risks (Edmondson, 2019). Furthermore, accepting various perspectives and even vastly differing viewpoints signal the acceptance of diversity and inclusion, which are associated with the successful implementation of innovative projects (Dokko et al., 2014; Newman et al., 2017). The project also improved team dynamics related to psychological safety and group cohesion (Appelbaum et al., 2019). These factors improve collaboration, knowledge sharing, and innovation within and between departments.

Interpersonal conflicts in the workplace can drain emotional resources, disrupt collaborative efforts, and hinder performance (Jehn and Mannix, 2001; Delice et al., 2019). Although our study showed the conduct of the project without interpersonal conflicts, preexisting conflicts could reignite. Nevertheless, shared focus and proactive collaboration can override these conflicts. The suggested conflict management strategies, such as focusing on tasks at hand and maintaining politeness and courtesy, are supported by research on effective strategies to mitigate the negative impacts of conflict (Tjosvold, 2008). In essence, the project served as a catalyst for positive change in the workplace by enhancing collaboration, managing conflicts, and improving team dynamics.

### 4.3. Leadership style and support

The theme of “Leadership Style and Support” highlights the foundational role of leadership in fostering innovation. The counselors’ experiences demonstrate how proactive, supportive leadership, and allocating resources toward the project facilitated the development and implementation of the innovation. These results agree with research suggesting that leadership behaviors, such as providing support and resources, can significantly influence innovation outcomes (Amabile et al., 2004; Kovach, 2020). The counselors’ reflections also highlight the importance of trust and acceptance in the leader-follower relationship. The project leader’s perception of gaining the trust and acceptance of the team suggests the presence of transformational leadership behaviors, which have been associated with increased innovation (Bass and Riggio, 2005; Lee and Hidayat, 2018). In turn, counselors’ appreciation of the leadership’s proactive and supportive role further underscores the importance of leadership in creating an environment conducive to innovation. Interestingly, the theme also suggests that leadership support may require a formal position in the organization. It may not be possible to overlook the role of power dynamics in influencing workplace innovation processes and outcomes (Kovach, 2020).

### 4.4. Personal reflections of the project leader

As the project leader and first author, I played a pivotal role in initiating and developing the project. Drawing on my previous experience with research projects in large organizations, I was acutely aware of the anonymity often felt by workers. My aim was to enhance the visibility of the counselors. I also recognized some internal tensions, which are common in any workplace. The tensions were primarily regarding certain aspects of providing services to specialized foster parents and differences of opinion among the counselors. My hypothesis was that a shared project would channel these energies toward a common goal and foster cooperation.

During the initial phase, I engaged with the team to understand their experiences, needs, and aspirations. This period of observation served as a form of reflexivity, allowing me not only to introduce myself, but also to engage in professional self-evaluation. I was deeply gratified by the respect and trust that the counselors gave me. This warm reception empowered me, fueling my motivation to invest in a project that would make counselors proud and visible within our large organization.

When I proposed the idea of an innovative training program, it received full support from both the leadership and the counselors. Achieving this level of buy-in within just a few months of my new role gave me a profound sense of inclusion and acceptance. This psychological safety encouraged me to take risks, a crucial element in innovative project work that aligns with existing literature on the importance of diversity and inclusion.

I was particularly encouraged to see my colleagues engage and stretch beyond their comfort zones. Strategically, I emphasized the need to start from the top, secure leadership buy-in, and then work from the bottom up. This approach underscores the vital role of proactive leadership in fostering an environment conducive to innovation. However, I also encountered skepticism and resistance regarding the scope of the project, its ultimate value, and the resources required to undertake the enterprise. It illustrated to me the complexity of bottom-up initiatives in large organizations.

The project allowed me to connect with my colleagues, leverage my background to promote their competencies, and enhance their visibility. Interestingly, this focus also had a reciprocal effect, reinforcing my own sense of competence and expertise.

## 5. Strengths and limitations

Several aspects of the study are methodological strengths. First, the interviews include all the counselors, department leaders and the project leader who developed and conducted the project. Therefore, the complete data is represented in the article. Second, as lead and second authors, both of whom were also employees, our insider perspectives during the project’s development enriched our understanding of the project’s context and dynamics. However, we are mindful of the possible diversity in our interpretation of the findings. The third author, an independent researcher, provided an external perspective, contributing to the triangulation of our data interpretation. We maintained reflexivity throughout the research process. For instance, the categories and subcategories reflect discipline-bound understanding, since the first and third authors are clinical psychologists. The categories also reflect the second author’s discipline-bound understanding as a clinical social worker and family therapist, especially conflict and social dynamics, which can be understood systemically.

We also acknowledge the limitations. First, the description and results of this study are deeply rooted in the specific context of the project within the Stavanger Department. Although this provides detailed and nuanced insights, it may limit the generalizability of the findings to other contexts or settings. Although steps were taken to reduce bias, introduce objectivity, and balance power imbalance, we acknowledge that the dual role of the first and second authors may limit the validity of lessons learned from this study. The shared background and experiences of the authors may have influenced the kind of responses that were considered valid and interesting. They may have also been more inclined to focus on data that confirmed their shared goals and perspectives, possibly overlooking contradictory data. Second, the study captures the experiences and perceptions of the participants at a specific point in time. Changes in the project, the work environment, or the broader social and organizational context over time may influence the experiences and perceptions of the participants. Lastly, the perspectives of other stakeholders, such as foster parents or other staff not directly involved in the project, have incorporated and may provide additional valuable insights. Nonetheless, future research could build on the knowledge shared by the counselors and proactively engage and track perspectives from the stockholders, including the vulnerable foster children, whom we wish to be the ultimate beneficiaries of the work.

## Data availability statement

The raw data supporting the conclusions of this article will be made available by the authors, without undue reservation.

## Ethics statement

Ethical approval was not required for the studies involving humans because the project was categorized as a quality assurance initiative and did not formally require consent. The studies were conducted in accordance with the local legislation and institutional requirements. The participants provided their written informed consent to participate in this study.

## Author contributions

PI: Conceptualization, Formal analysis, Investigation, Methodology, Project administration, Resources, Writing – original draft, Writing – review & editing. JR: Investigation, Validation, Writing – review & editing, Formal analysis, Methodology. JB: Formal analysis, Methodology, Validation, Writing – review & editing.
